# Current Treatment Approaches to HCC with a Special Consideration to Transplantation

**DOI:** 10.1155/2016/7926264

**Published:** 2016-06-20

**Authors:** N. Bhardwaj, M. T. P. R. Perera, M. A. Silva

**Affiliations:** ^1^Department of Hepatobiliary and Pancreatic Surgery, Churchill Hospital, Oxford University Hospitals NHS Trust, Oxford OX3 7LE, UK; ^2^The Liver Unit, University Hospital Birmingham NHS Foundation Trust-Queen Elizabeth, Birmingham 15 2TH, UK

## Abstract

Hepatocellular carcinoma (HCC) is the third leading cause of cancer deaths worldwide. The mainstay of treatment of HCC has been both resectional and transplantation surgery. It is well known that, in selected, optimized patients, hepatectomy for HCC may be an option, even in patients with underlying cirrhosis. Resectable patients with early HCC and underlying liver disease are however increasingly being considered for transplantation because of potential for better disease-free survival and resolution of underlying liver disease, although this approach is limited by the availability of donor livers, especially in resectable patients. Outcomes following liver transplantation improved dramatically for patients with HCC following the implementation of the Milan criteria in the late 1990s. Ever since, the rather restrictive nature of the Milan criteria has been challenged with good outcomes. There has also been an increase in the donor pool with marginal donors including organs retrieved following cardiac death being used. Even so, patients still continue to die while waiting for a liver transplant. In order to reduce this attrition, bridging techniques and methods for downstaging disease have evolved. Additionally new techniques for organ preservation have increased the prospect of this potentially curative procedure being available for a greater number of patients.

## 1. Introduction

Primary liver cancer in the form of hepatocellular carcinoma (HCC) is the fifth most common cancer and the third commonest cause of cancer deaths worldwide [1]. Over 3,200 new cases are registered in the UK each year [2] and although rare in the UK, the incidence of primary liver carcinoma is predicted to rise in the future as a consequence of the hepatitis C virus epidemic and alcohol and nonalcoholic steatohepatitis (NASH) [3–5]. Treatment of HCC can be classified into curative, palliative, and symptomatic. Curative treatment includes, surgery, transplantation, and local tumour ablation. Unfortunately due to either tumour stage, size, and/or anatomy, patient comorbidities, or shortage of donor livers from both cadaveric and living donors, only 20% of patients undergo curative treatment [6, 7]. The purpose of this review is to provide an update on the current role of transplantation in the treatment of HCC and the strategies employed to increase the donor pool, improve the overall survival of patients on the transplant register, and reduce the number of patients dropping out of the waiting lists. These include evaluation of extended criteria, the use of bridging therapies while patients are awaiting a transplant, the concept of allocation and prioritising, the use of living related donor transplants, the implications of using marginal grafts for HCC, and the role of downstaging therapies. Prior to that it is essential to provide a brief overview of the different staging systems proposed for HCC and the role of surgery in the treatment of HCC.

## 2. Staging

In up to 5% of cases in the west and 40% of cases in Asia, HCC develops on the background of normal liver parenchyma [8]. These patients are best treated with surgical resection and as the liver has normal regenerative capacity, they can often tolerate major liver resections (up to 70% of liver volume) [9] without significant morbidity, achieving a 5-year survival of 60–65% [10–12]. Of the several staging systems that exist, the 7th TNM edition in accordance with the AJCC fails to consider the patient's hepatic functional status [13]. Similarly Child-Pugh classification, although useful as a snapshot of degree of liver dysfunction, is one dimensional and does not allow for accurate pathological status to be accounted for. The Cancer of the Liver Italian Program (CLIP) classification [14] and the Chinese University Prognostic index (CUPI score) [15] tend to classify patients who have more advanced disease and are not descriptive enough to be able to distinguish those patients with lower volume of disease who may benefit the most from surgical intervention. The French Classification [16] and the Japan Integrated Staging (JIS), which has recently been refined to include biomarkers (AFP, DCP, and AFP-L-3) [17], are also staging systems but do not assign treatment allocation to specific prognostic subclasses as opposed to the Barcelona Liver Cancer Staging system (BCLC).

## 3. Surgical Resection

**Figure 1:**
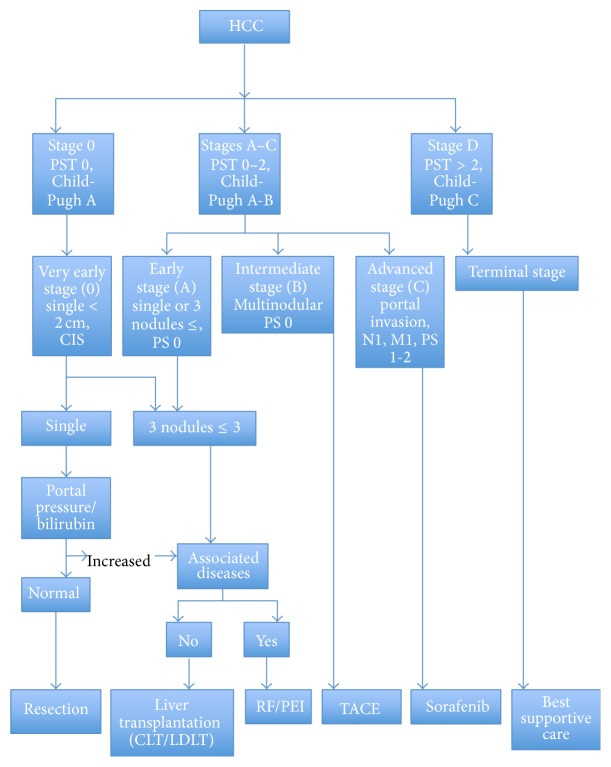
BCLC staging of HCC with treatment recommendations.

The BCLC is currently the commonest staging system used in patients with underlying cirrhosis and is endorsed by the European Association for the Study of the Liver-American Association for the Study of Liver Diseases (EASL-AASLD) and the European Association for the Study of the Liver-European Organisation for Research and Treatment of Cancer (EASL-EORTC). The guidelines recommend surgical resection in all Child-Pugh A patients with a solitary very early stage HCC (<2 cm) [8, 27] (Figure 1). Early stage HCC is defined as per the Milan criteria as a single nodule ≤ 5 cm in diameter or ≤3 nodules ≤ 3 cm in diameter [28] and either local ablation in the form of radiofrequency or percutaneous ethanol injection or transplantation is recommended as per the guidelines. However, due to the paucity of donors or lack of access to transplant centres, many surgeons would undertake resection in these patients. This requires many factors to be considered and these can be divided into tumour (size, number, and location), patient (performance status, comorbidities), and liver (functional reserve and degree of portal hypertension and portal vein involvement). Theoretically tumour size and number are irrelevant as long as adequate clearance, ideally an anatomical resection with 1 cm resection margin, is achieved without compromising residual liver function [29–31]. There is evidence from a large multicentre trial that 30- and 90-day mortality rates for liver resections outside the EASL-AASLD guidelines between stage 0-A, B, and C were not significantly different and the overall 5-year survival for stage 0-A, B, and C patients was 61%, 57%, and 38%, respectively. At multivariate analysis, bilirubin, size (>5 cm), macrovascular invasion, cirrhosis, and oesophageal varices were independent predictors of poor survival [32]. There are reports from large volume centres that have resected multinodular HCCs and all patients with multinodular disease had poorer overall survival compared to single lesions [33–35]. Importantly however all groups commented on the survival rate of these patients still being better than in those patients not offered curative treatment [36].

## 4. Transplant Criteria

Liver transplantation as treatment for HCC is extremely attractive as it guarantees complete resection of the tumour and also removes potentially preneoplastic lesions and undetected multifocus disease. In addition, it addresses the underlying cirrhosis, thus negating the potential risk of developing cirrhosis related problems in the future such as portal hypertension, liver failure, and recurrent HCC. Transplantation for HCC was associated with extremely poor prognosis with early recurrence and poor long-term survival [37, 38] till the introduction of the Milan criteria by Mazzaferro et al. in 1996 [28]. The overall actuarial survival at 4 years was 75% and the recurrence-free survival was 83% in this landmark study. They further justified their criteria by publishing a meta-analysis in 2011 that comprehensively validated the Milan criteria's ability to capture tumours with favourable biology and hence improved survival [39]. The Milan criteria have also been independently validated by several studies [40–42] and widely adopted in USA by the United Network of Organ Sharing (UNOS) [43]. However, in response to concerns that the Milan criteria were perhaps too restrictive and potentially excluding patients who may benefit from liver transplantation, a group of surgeons embarked on operating on patients outside the Milan criteria, the so-called “extended criteria.”

## 5. Extended Criteria

The University of California, San Francisco (UCSF) proposed a modest expansion of the Milan criteria to include patients with single lesion up to 6.5 cm or up to 3 lesions none larger than 4.5 cm and a tumour volume no greater than 8 cm. They concluded that this extended criteria did not adversely affect 5-year survival, which they reported at 75.2% [18]. They further validated the UCSF expanded criteria and suggested that they predicted survival as accurately as the Milan criteria and could serve as selection criteria for liver transplant [22]. A recent study also suggested that the survival between the UCSF criteria and the Milan criteria was comparable [44]. The UCSF criteria are the only extended criteria that have been validated independently on either explant pathology or radiology [45–48].

A group from Pamplona, Spain, expanded their transplant criteria to include patients with one lesion up to 6 cm or 2-3 lesions, none greater than 5 cm, and reported a 5-year actuarial survival of 79% [19]. A group from Mt. Sinai reported that tumours up to 7 cm could be transplanted with 5-year survival in the region of 55%, although this group used a combination of neoadjuvant treatment of systemic chemotherapy and chemoembolisation [20]. A group from Edmonton compared patients undergoing transplant meeting the Milan criteria with those meeting their extended criteria (1 tumour < 7 cm or any number < 5) and reported a 4-year recurrence-free survival of 81% and 76% in the Milan and extended criteria, respectively [21]. Similarly a group from Houston reported a 5-year survival of 70.2% in their extended criteria (1 lesion < 6 cm, ≤ 3 lesions none > 5 cm, total diameter 9 cm) [26]. A large retrospective multicentre study analysed 1556 patients across 36 centres and suggested that, in patients with an “up-to-seven” criteria (sum of the number of nodules and diameter of the largest tumour in cm does not exceed seven) with no macrovascular invasion (MVI) and extrahepatic spread (EHS), a five-yr survival rate of 71.2% can be achieved. [25]. This study also worked on the concept of a metroticket, whereby clinicians can estimate 5-year survival based on the number of tumours, the size of the largest tumour, and the presence of vascular invasion (Table 1).

The UK listing criteria were based on the Milan criteria up until 2009, after which they were expanded and the current guidelines are a single tumour no greater than 5 cm in diameter or up to 5 tumours all no greater than 3 cm or a single tumour greater than 5 cm but not greater than 7 cm with no evidence of tumour progression (volume increase < 20%) and no extrahepatic spread and no new nodule formation over a 6-month period. Tumour rupture and an AFP > 10,000 are absolute contraindications as are extrahepatic spread and macroscopic vascular invasion [49]. The criteria have not been validated and some argue that a maximum tumour diameter of 15 cm is well above the Milan and UCSF maximum tumour guidelines. A maximum tumour size greater than 7 cm is associated with poor survival [50] as is a total tumour size (sum of diameters) of 10 cm or larger, which in a meta-analyses was associated with four times increased risk of death or recurrence [51]. In addition some argue that a volume increase of <20% is difficult to measure on scans and is ambiguous [52]. Another criticism of the UK listing criteria is that the value of AFP > 10,000 is too high, particularly as there is robust evidence that an AFP of >1000 ng/mL is associated with vascular invasion and poor tumour differentiation and hence poorer outcome [53].

## 6. Allocation and Prioritising

Although difficult to truly asses, the estimated drop-out of patients waiting on the transplant list ranges from 10 to 15% in the US and up to 35% in Europe [7]. Thus, the impetus to increase donor pool and diminish the tumour progression rate led the United Network for Organ Sharing in 2002 to incorporate the Model for End Stage Liver Disease (MELD), which was originally generated to predict 3-month survival in patients with End Stage Liver Disease [54], into a new scoring system. Since the implementation of this new policy, several studies have shown an improvement in waiting time to transplant and number of patients transplanted, an increase in 5-month waiting list survival, and a reduction in the drop-out rate compared to the pre-MELD era [55, 56]. The updated version of this scoring system assigns no points to tumours < 2 cm but 22 points (or an initial MELD score equivalent to 15% 3-month mortality) to single tumours between 2 and 5 cm or up to 3 nodules each < 3 cm [57] as there was no evidence that T1 tumours (<2 cm) had an increased risk of drop-out and some studies suggested that the initial UNOS criteria were prioritising early tumours unnecessarily [58]. The latest policy has been validated and concluded that the reduced MELD priority score does not adversely impact on patient survival [59]. The UK End Stage Liver Disease (UKELD) does not assign extra points to patients with HCC [60] and as a consequence patients may be at an increased risk of drop-out compared to their counterparts in the US.

## 7. Bridging Therapy

In order to minimise drop-out of patients while awaiting transplant, which is estimated to be almost 30% [60], several strategies have been developed, including Radiofrequency Ablation (RFA), Transarterial Chemoembolisation (TACE), and surgical resection [61]. There are no randomised controlled trials and the potential benefits of using locoregional therapies are based on observational and cost-analysis studies. Several studies have shown the efficacy of RFA in controlling the progression of HCC [62–67] and subsequently reducing the drop-out rate to below 25% [62, 68]. TACE delivers a two-pronged attack on the HCC lesion by combining an ischaemic insult with cytotoxic drugs and has been shown to be effective in preventing tumour progression in patients meeting the Milan criteria on the waiting list [69, 70].

Surgical resection followed by transplantation does not increase surgical risk nor impair survival [71] and a recent systematic review of 16 studies found that, of those 7 studies which reported salvage transplantation rates, the median rate of salvage transplantation was 41% after a median time to recurrence of 21 months. It concluded that salvage liver transplant following primary hepatic resection has 5-year survival of 67%, which is comparable to upfront liver transplantation [72]. These results were also replicated by another meta-analysis which although did report increased operating time and blood loss in those patients undergoing salvage transplantation compared to primary transplantation, reported no difference in postoperative morbidity or perioperative mortality, length of hospital stay, or 5-year survival between the two approaches [73]. These studies suggest that salvage liver transplant is a viable strategy for those patients waiting for a liver transplant.

## 8. Living Donor Liver Transplant

The vast majority of patients in the west receive a deceased donor liver transplant (DDLT); however there is increasing interest in the use of living donor liver transplant (LDLT) as a possible means of increasing the donor pool. Less than 5% of adult liver transplants use a living donor, which is in stark contrast to kidney transplantation, where living donors comprise 40% of all cases performed [74]. The disparity can be explained by the life threatening complication risk of up to 2% and a mortality rate of up to 0.3% associated with a donor hepatectomy [74–76]. Using a decision analytical model taking into account the risk of drop-out while waiting (4% per month), the expected survival of the recipient (70% at 5 years), and the risk for the donor (0.3% mortality), it has been reported that patients with HCC waiting more than seven months for a DDLT would benefit from a LDLT [77]. Initial concerns of LDLT being associated with higher recurrence rates, due to the “fast-track” effect, have been unfounded [78] and LDLT recurrence and survival rates are comparable to DDLT with lower waiting times [79]. Two meta-analyses reported similar overall survival between LDLT and DDLT; however one found LDLT to associated with reduced disease-free survival [80] while the other found similar survival outcomes in both groups with no increase in HCC recurrence in the LDLT group [81]. Currently the EASL-EORTC recommends restricting LDLT to centres of excellence in hepatic surgery and transplantation and does not recommend LDLT to be used in the context of extended criteria [8].

## 9. Downstaging

There have been reports to suggest that patients initially beyond the Milan criteria can be downstaged with the use of locoregional therapies (TACE or TACE ± RFA) and transplanted with excellent results [82–84]. However, there is lack of consensus as to which patients should be considered for downstaging, the modality most suitable for downstaging, and criteria by which successful downgrading should be considered. A recent systematic review reported 8 observational studies, mostly prospective, and found a wide variety of inclusion criteria, no uniform locoregional treatment regimen, and no standardised way of reporting successful downstaging [85]. Similarly a systematic review suggested selective internal irradiation with yttrium-90 microspheres as a tumour downstaging treatment or a bridge to transplantation [86]; however its use has not been widely accepted. Nevertheless patients downstaged to within the Milan criteria achieved comparable overall survival and disease-free survival after liver transplant to those patients who met the Milan criteria and the authors argue that more patients should be considered for downstaging. It is often argued that patients should not be denied a transplant based on size and number of tumours and instead tumour biology as expressed by the degree of microvascular invasion and tumour grade, known predictors of survival and recurrence, should play a greater role in the decision making process. Hence, the authors recommend a preoperative biopsy in all patients being considered for downstaging. The EASL-EORTC recommend downstaging only those patients already on the waiting list for a transplant whose tumours progress beyond the Milan criteria [8].

## 10. Marginal Grafts

Donation after cardiac death (DCD) can be a potential source of transplantable organs and thus expand the donation pool. Recent report suggests that DCD donors are used more frequently than donation after brain death (DBD) with the result that in the past decade in the US there has been a greater than 100% increase in DCD donations [87–89]. However, DCD allografts are associated with higher rates of graft failure, biliary complications, and reduced survival [90]. Warm and Cold Ischaemic Time (WIT/CIT) are critical in graft survival in DCD donors. Several measures, including judicious donor selection, donor age below 40 years, and no steatosis and a specific resuscitation technique, including preservation of the organ with systemic heparin, the use of extracorporeal oxygenation, a short WIT of less than 15 min, and a short CIT (less than 10 h), have shown to reduce primary nonfunction and biliary complications [91, 92]. More recently, normothermic machine perfusion has been proposed as a technique used to maintain donor organs in a physiological state and even resuscitate grafts, avoiding the depletion of cellular energy and the accumulation of waste products, which occurs with static cold storage [92]. This method also enables viability assessment prior to transplantation thereby reducing the risk of transplanting inherently marginal organs [92]. Treatment of DCD livers with hypothermic oxygenated perfusion improved 1-year graft survival and significantly reduced graft injury [93] and similarly another study suggested two hours of oxygenated hypothermic machine perfusion after traditional static cold storage restores hepatic ATP levels and improves hepatobiliary function but does not reduce (preexisting) hepatobiliary injury in extended criteria livers [94]. It is likely therefore that these techniques will increase the donor pool and results from further trials in liver transplantation are eagerly awaited.

## 11. Discussion

Outcomes following treatment of HCC improved dramatically following the introduction of the Milan criteria by Mazzaferro et al. in 1996 [28]. Prior to this, results following liver transplantation for HCC were on the whole dismal. On the back of this success, subsequent studies have relentlessly challenged the perceived restrictive nature of the Milan criteria. Of these only the UCSF criteria have been validated independently although currently liver transplantation is offered to patients even beyond the UCSF criteria.

Downstaging and bridging therapy have further improved outcomes ensuring a larger number of patients under liver transplantation with acceptable outcomes. With the criteria for listing a patient for liver transplantation becoming more standardised, more patients became eligible for liver transplantation. This has resulted in a shortage in donor organs and invariable attrition of patients on transplant waiting lists. With the emergence of DCD donors and refinement of criteria for the use of such organs, along with the addition of exiting new techniques for donor organ resuscitation, the donor pool continues to grow. This increase in opportunity availing patients with HCC a liver transplant is likely to make an impact of the similar magnitude the Milan criteria did in the late 1990s on the outcome of treatment for HCC.

## Figures and Tables

**Table 1:** Comparison of survival; Milan versus extended criteria.

Study	Number	Milan criteria	Extended criteria	5-year survival	
				Milan	Extended
Yao et al. [18]	60	46	14	72%	72%

Herrero et al. [19]	61	49	12	79% entire cohort	

Roayaie et al. [20]	31	0	31	N/A	55%

Kneteman et al. [21]	40	19	21	4-year survival	
				87%	83%

Yao et al. [22]	168	130	38	80%	82%

Onaca et al. [23]	1152	1038	114	62%	54%

Cillo et al. [24]	100	60	40	3-year survival	
				69%	85%

Mazzaferro et al. [25]	1556	444	1112	Up-to-seven	
				73.3%	71.2%
				Exceeding up-to-seven	
				73.3%	53.6%

Guiteau et al. [26]	445	363	82	72.9%	70.2%
